# Gardner Fibroma Presenting as a Suspected Encephalocele: A Case Report and Literature Review

**DOI:** 10.7759/cureus.92558

**Published:** 2025-09-17

**Authors:** Abdelkouddouss Laaidi, Karim Baayoud, Chaimaa Amry, Oufaa Jamal, Khadija El Guettabi, Said Hilmani, Abdessamad Naja, Abdelhakim Lakhdar

**Affiliations:** 1 Neurosurgery, University Hospital of Casablanca, Casablanca, MAR; 2 Neurosurgery, Centre Hospitalier Universitaire Souss Massa d'Agadir, Agadir, MAR; 3 Neurosurgery, Ibn Rochd Hospital Center, Casablanca, MAR

**Keywords:** encephalocele, familial adenomatous polyposis (fap), gardner’s fibroma, soft tissue tumor, surgical excision and prognosis

## Abstract

Gardner fibroma (GF) is a rare benign tumor of soft tissues, most frequently described in children and adolescents, and occasionally representing an early manifestation of familial adenomatous polyposis. We report a case of a 14-month-old infant with no relevant medical history who presented with a midline occipital mass. The lesion was well-epithelialized and non-fistulized, with no evidence of pus, cerebrospinal fluid leakage, or inflammation, and measured 7 × 7 × 8 cm. Neurological examination was normal. Computed tomography revealed a heterogeneous, hypodense soft tissue mass with punctate central calcifications. The patient underwent gross total resection; the tumor appeared white, solid, and cartilaginous, adherent to the dura with an underlying bone defect, but without significant vascularity. Histopathological analysis confirmed the diagnosis of Gardner fibroma. The postoperative course was uneventful. At two months of follow-up, the patient remained asymptomatic, except for delayed language development.

This case may represent one of the first documented instances of cranial GF presenting as an encephalocele-like lesion. A review of the literature shows that GF typically arises in the paraspinal or shoulder regions, and its recognition in rare cranial sites is clinically significant, as it may represent the earliest manifestation of an inherited cancer predisposition syndrome. When assessing cranial masses, Gardner fibroma should be considered in the differential diagnosis alongside encephalocele and dermoid cyst. Complete surgical excision remains the treatment of choice to minimize recurrence.

## Introduction

Gardner fibroma (GF) is a rare benign soft tissue lesion most commonly encountered in children and adolescents. Its clinical significance lies in its frequent association with familial adenomatous polyposis (FAP) and Gardner syndrome [1,2]. Although GF usually presents as a slow-growing, painless mass, diagnosis can be challenging when the tumor arises in atypical sites, such as the cranium. In these locations, GF can mimic other cranial pathologies, including encephalocele, which is defined as the herniation of intracranial contents through a congenital or acquired skull defect [3].

Here, we describe a unique case of cranial GF in a pediatric patient who was initially suspected to represent an encephalocele based on preoperative findings. We could not identify earlier reports of cranial GF in the sources reviewed. Misdiagnosis in such cases may lead to inappropriate surgical planning or delayed recognition of an underlying predisposition to FAP. We also review current management strategies for GF, emphasizing the importance of histopathological confirmation and complete surgical excision to minimize the risk of recurrence.

## Case presentation

A 14-month-old male infant with no significant medical history was referred for evaluation of a midline occipital mass present since birth. According to the parents, the mass had gradually increased in size over the first year of life. On admission, he was alert, afebrile, and in good general condition, with a head circumference of 50 cm, a weight of 11 kg, and a length of 80 cm, all within normal ranges for his age.

Physical examination revealed a well-epithelialized, non-fistulating occipital mass measuring 7 × 7 × 8 cm, without evidence of pus, cerebrospinal fluid (CSF) leakage, or local inflammation. Neurological assessment demonstrated age-appropriate psychomotor development, with no focal deficits.

**Figure 1:**
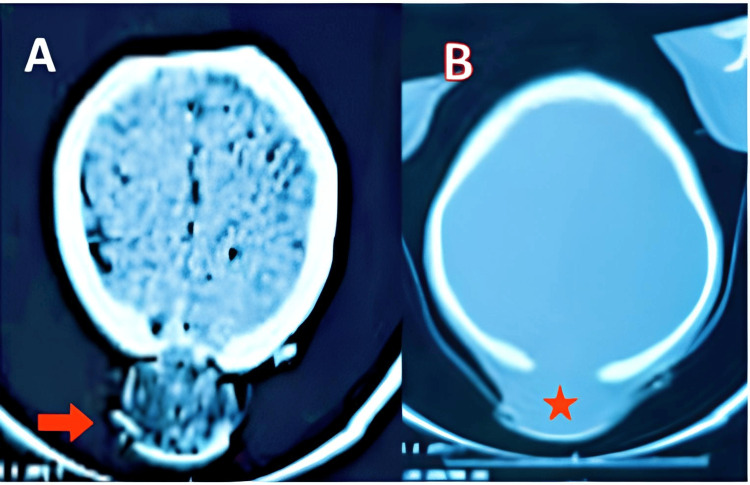
Cerebral CT scan in axial sections. Parenchymal window (A) and bone window (B). The images show (A) an extra-axial occipital lesion, rounded, slightly hypodense with central punctate hyperintensities (red arrow); and (B) occipital bone defect underlying the lesion (red star).

A preoperative computed tomography (CT) scan revealed a heterogeneous, hypodense occipital lesion containing punctate hyperdensities, communicating across an underlying bone defect between intracranial and extracranial compartments (Figures 1A, 1B). An associated mega cisterna magna was also noted. MRI was considered to better delineate intracranial involvement; however, it was not performed due to resource limitations, and the CT findings were deemed sufficient for surgical planning.

**Figure 2:**
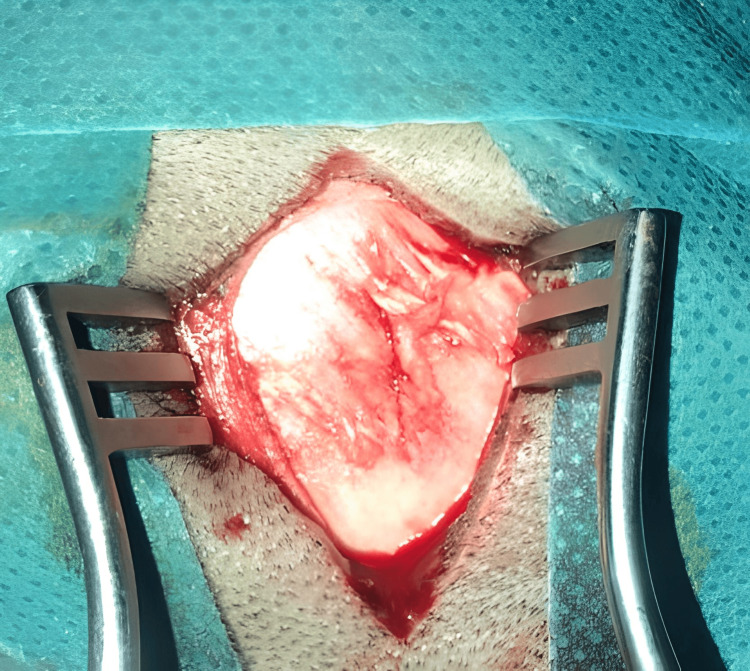
Intraoperative appearance; white, avascular mass with cartilaginous consistency.

Given the aesthetic concern and risk of complications from the bone defect, complete excision was performed. Intraoperatively, the lesion appeared as a white, avascular mass with cartilaginous consistency, firmly adherent to the dura mater (Figure 2). The dura was carefully preserved during dissection, with no breach or cerebrospinal fluid (CSF) leakage. Gross total resection was achieved, followed by layered closure.

**Figure 3:**
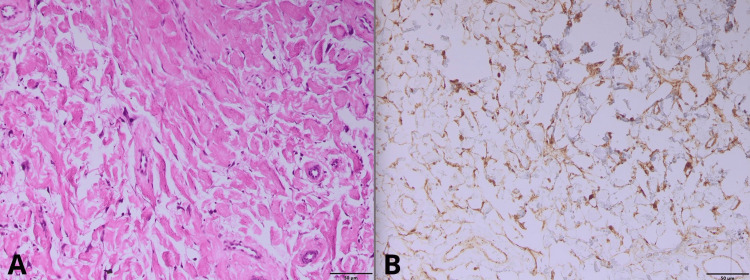
Gardner fibroma: histopathological (A) and immunohistochemical (B) features.

Histopathological examination revealed dense collagen bundles arranged in haphazard fascicles, with scattered bland fibroblasts and no atypia. Immunohistochemistry demonstrated diffuse CD34 positivity and nuclear β-catenin staining, confirming the diagnosis of Gardner fibroma (Figures 3A, 3B).

A postoperative CT scan confirmed gross total resection of the lesion with no evidence of hemorrhage or other complications. The patient was referred for genetic counseling and testing for familial adenomatous polyposis; however, testing has not yet been performed. At two months follow-up, neurological examination was normal aside from mild language delay, likely unrelated to the surgical intervention. Long-term follow-up, including regular clinical and radiological assessment, is planned.

## Discussion

Gardner fibroma (GF) is a rare benign soft-tissue tumor with distinct histological features, including disorganized fascicles of coarse, hyalinized collagen bundles, a hypocellular stroma with bland spindle-shaped fibroblasts, absence of atypia or necrosis, and sparse vasculature. Immunohistochemically, tumor cells show CD34 positivity and nuclear β-catenin expression, corroborating the diagnosis. Frequently, germline APC mutations explain its association with familial adenomatous polyposis (FAP) and Gardner syndrome [3-5].

Imaging findings can raise suspicion but remain non-diagnostic. Ultrasound typically reveals a well-circumscribed hyperechoic or isoechoic mass, representing its collagen-rich matrix [3]. On CT, GF appears iso- to hypoattenuating with minimal contrast uptake; in this case, the lesion exhibited central hyperdensities and an underlying bony defect communicating with intracranial compartments, mimicking an encephalocele [3,6]. MRI generally shows low signal on both T1 and T2 sequences and mild, variable enhancement due to collagen content, limiting specificity [3,6]. These imaging features merit cautious interpretation and correlation with histopathology.

The cranial location in the present case initially suggested encephalocele, characterized by herniation of cerebrospinal fluid (CSF) or brain tissue through a skull defect, a major diagnostic consideration in pediatric neurosurgery. Other entities to consider include dermoid/epidermoid cysts, desmoid tumors, and low-grade sarcomas, which can present similarly on imaging [7].

A comprehensive clinicopathologic series involving 45 patients with 57 Gardner fibromas demonstrated that these lesions predominantly occur in extracranial soft tissues as follows: notably, the paraspinal region (61%), followed by the head and neck (14%), extremities (14%), and chest or abdominal wall (11%) [8,9]. Although GF typically presents in these locations, rare and atypical sites have been reported, such as a floor-of-mouth lesion in a 10-year-old boy, and a retropharyngeal GF causing upper airway obstruction in a 16-month-old infant [10,11]. A review of the literature revealed no prior descriptions of a cranial GF mimicking an encephalocele, suggesting the importance of the current observation.

Surgical management of GF requires complete en bloc resection to minimize recurrence risk, especially given potential progression to desmoid fibromatosis [12]. In our patient, gross total resection was achieved without complications, and postoperative imaging confirmed no residual disease. Given the unpredictable biological behavior of Gardner fibroma, including its potential for progressive growth, long-term clinical surveillance remains essential [2].

This fibroma can serve as an initial clue to the diagnosis of Gardner syndrome, characterized by polyposis coli, osteomas, desmoid tumors, extracolonic cancers, and various soft-tissue tumors. Multidisciplinary follow-up for the development of colorectal tumors, as well as a familial screening for FAP, is recommended [12,13].

For pediatric neurosurgeons, awareness of this rare mimic is critical. Misdiagnosing GF as an encephalocele may lead to unnecessary or suboptimal surgical approaches. Accurate preoperative interpretation, pathologic correlation, and interdisciplinary collaboration are necessary to guide appropriate management.

## Conclusions

Gardner fibroma, though rare, should be considered in the differential diagnosis of cranial masses in infants and children. Accurate diagnosis relies on histopathological examination. Complete surgical excision is essential to prevent recurrence. Given the potential association with familial adenomatous polyposis, genetic evaluation and appropriate surveillance are recommended for affected patients and their families.
